# Analysis of the Influence of Different Settings of Scan Sequence Parameters on Vibration and Noise Generated in the Open-Air MRI Scanning Area †

**DOI:** 10.3390/s19194198

**Published:** 2019-09-27

**Authors:** Jiří Přibil, Anna Přibilová, Ivan Frollo

**Affiliations:** Institute of Measurement Science, Slovak Academy of Sciences, 841 04 Bratislava, Slovakia; anna.pribilova@savba.sk (A.P.); umerollo@savba.sk (I.F.)

**Keywords:** magnetic resonance imaging, mechanical vibration, acoustic noise, signal processing

## Abstract

A system of gradient coils of the magnetic resonance imaging (MRI) device produces significant vibration and noise. Energetic relations of these phenomena are analyzed depending on MRI scan parameters (sequence type, repetition time (TR), echo time (TE), slice orientation, body weight). This issue should be investigated because of negative physiological and psychological effects on a person exposed to vibration and acoustic noise. We also measured the sound pressure level in the MRI scanning area and its vicinity in order to minimize these negative impacts, depending on intensity and time duration of exposition. From the recorded vibration and noise signals, the energy parameters were determined and statistically analyzed, and the obtained results were visually and numerically compared. Finally, subjective evaluation by a listening test method was used to analyze the influence of the generated MRI noise on the human psyche.

## 1. Introduction

Magnetic resonance imaging (MRI) is an effective method for structure investigation of biological samples [1] or human body parts, such as a head [2,3], a thorax [4], etc. In addition to the standard closed-bore MRI scanner, the open-air one is increasingly used in special cases, e.g., in claustrophobic patients [5]. In this MRI device, two parallel permanent magnets form a static magnetic field between them [6]. A gradient system consisting of two internal planar coils parallel to the magnets is used to select slices in three dimensions. A tested object is placed in the magnetic field, together with an external radio frequency (RF) receiving/transmitting coil.

The coil current changes quickly during gradient switching, resulting in undesirable vibration of the whole structure [7], and subsequent acoustic noise disturbing the speech recorded during articulation and concurrent three-dimensional (3D) MRI scanning for examination of dynamic changes in the shape of the vocal tract and vocal folds [8]. In this case, a speech denoising method must be applied on the recorded signal. Another solution includes the recording of a speech signal by a special fiber optical microphone that can be located in the MRI scanning area [9]. Here, real-time speech processing is enabled during MR scanning at the expense of rather complicated practical realization involving synchronization of both processes, special hardware for an MRI device, etc. A cheaper type of an optical microphone (e.g., the first or the second generation of the Optoacoustics FOMRI microphone) has a limited frequency response in the range between 50 and 4000 Hz that is insufficient for our purpose. The third generation of the Optoacoustics FOMRI microphone system using fiber-optic technology and active noise reduction [10] can solve this problem; however, this solution is more expensive.

Acoustic noise interference is not only a technical problem, but a physiological and psychological one as well. These negative effects on humans are well known in industrial environments with long-term noise exposure [11]; however, they evolve gradually and can be observed first on a short-term scale. The intensity of the vibration and the resulting acoustic noise and the time duration of its exposure are crucial factors affecting the degree of their physiological, as well as psychological impact. 

Motivation of our work was an exploration of energetic relations between the vibration and the noise in the scanning area of the low-field open-air MRI device and its surroundings to find a proper choice of a scan sequence and its parameters—sequence type, repetition time (TR), echo time (TE), slice orientation, etc. In addition, the volume inserted in the scanning area influenced the intensity of the vibration and noise produced in the scanning area of the MRI device. Finally, a tested object with its mechanical properties caused the change in the overall mechanical impedance when it loaded the system of lower gradient coils placed in the patient’s bed.

The experimental part has the following structure: In the preliminary phase, the sensitivity and the frequency response of the used vibration sensor were determined. Time-domain vibration and noise signals were recorded and processed, along with the measured sound pressure level (SPL), to find energetic features that were then evaluated statistically. Next, analysis of the influence of scan parameters on the time duration of the executed MR sequence and on the quality factor of the obtained MR images was carried out. Finally, the energetic features determined from the measured data were compared with the results of the subjective evaluation based on the listening test method.

## 2. Subject and Methods

As the open-air MRI is used primarily in medical practice, planes perpendicular to three axes of the Cartesian coordinate system are called according to medical terminology [12]. Anterior and posterior parts of a human body are divided by a frontal (coronal) plane. Another vertical plane divides a body to its left and right sides and is called a sagittal plane. The third plane dividing a body horizontally into superior and inferior parts is called a cross-sectional (transverse) plane. The scan orientation is selected by activation of the corresponding gradient coils, so the current flowing through them, as well as the resulting vibration and acoustic noise during execution of the scan sequence, depend on this selection. Two of the fundamental pulse sequences are preferably used in this MRI device: A spin echo (SE), being an excitation pulse followed by one or more refocusing pulses, and a gradient echo (GE), produced by conjunction of an excitation pulse with a gradient field reversal [13]. For optimal operation of the MRI unit, different parameters of the used scan sequence (the field of view, the number of slices, the thickness of a slice, etc.) are used for different scanned objects. The intensity of the vibration and noise produced by the MRI system depends not only on the setting of these parameters, but on the volume and the weight of the examined object as well. The tested object (a person, a sample, or a phantom) loads the lower gradient coil structure and thus becomes a part of the mechanical vibration system with its mass, stiffness, and damping.

If the vibration is to be picked up while the MRI sequence is executed, the vibration sensor placed in the MRI scanning area must not contain any ferromagnetic part to prevent its interaction with the static magnetic field, which may decrease the quality of the acquired image. It is essential that the sensor has good sensitivity with as small a dependence on frequency as possible. Using the reference sensitivity *B*_a0_ at a chosen reference frequency, the frequency-dependent sensitivity (frequency response) of an accelerometer *B*_a_ [mV/ms^−2^] may be expressed in [dB] by a relation:

(1) $$G_{a\log}\left(f\right)=20\cdot \log_{10}\left(B_{a}\left(f\right)/B_{a0}\right).$$

The sensor’s frequency range should cover harmonic frequencies of the vibration and noise signals concentrated in the low band due to limited frequency spectrum of the gradient pulse sequence. These requirements can be fulfilled by sensors constructed for acoustic pickup in musical instruments, or other ones based on the piezoelectric principle. First usage of all these vibration sensors must be preceded by measurement of their sensitivity and frequency response.

For obtaining high-quality MR images without artifacts, the acoustic sensors (the measuring microphone and the sound level meter) containing ferromagnetic parts must be placed beyond the influence of this static magnetic field, although, adequately close to the noise source. The sensitivity of the recording microphone and its directional pattern are selected in regard to effective rejection of the ambient noise. The sound level meter enables choice of the type of frequency weighting to match human perception of silent sounds (A weighting with more suppressed low and high frequencies) and loud sounds (C weighting with much less suppression of low frequencies than A weighting). Due to high measured sound pressure levels, we chose C weighting.

Several approaches can be applied for determination of the signal energy. Three of them represent the basis of our comparisons:

1. The standard root mean square (RMS) is calculated from a signal *x*(*n*) in a defined frame (window) with the length of *M* samples:
  (2) $$Signal_{RMS}=\sqrt{\frac{1}{M}\overset{M}{\underset{n=1}{\sum }}{\left|x\left(n\right)\right|}^{2}},$$
2. Another energetic parameter is determined from the Teager–Kaiser energy operator *O*_TK_ [14]:
  (3) $$O_{TK}=x{\left(n\right)}^{2}-x\left(n-1\right)\cdot x{\left(n+1\right)}_{}^{},En_{TK}=abs{\left(\frac{1}{M-2}\sum_{n=1}^{M-2}O_{TK}\left(n\right)\right)}_{}.$$
3. The third approach uses the short-term *N*_FFT_-point fast Fourier transform (FFT) to compute the power spectrum |S(k)|^2^, and in each frame, the energy is assessed from the first cepstral coefficient *c*_0_ or from the autocorrelation coefficient *r*_0_:
  (4) $$En_{c0}=\sqrt{{\left[\prod_{k=1}^{N_{FFT}/2}{\left|S(k)\right|}^{2}\right]}^{\frac{2}{N_{FFT}}}},En_{r0}=\frac{2}{N_{FFT}}\sum_{k=1}^{N_{FFT}/2}{\left|S(k)\right|}^{2}.$$

Next, the basic and supplementary spectral properties are determined from the recorded noise and vibration signals. Methods similar to those used in speech signal analysis can be applied for processing of these signals whose spectral content lies within the standard audio frequency range. The basic spectral properties (basic resonance frequencies *F*_V1_ and *F*_V2_ and their ratios, spectral decrease, etc.) are usually determined from the spectral envelope. The supplementary spectral features (spectral centroid (SC), harmonic-to-noise ratio (HNR), spectral entropy, etc.) describe the shape of the power spectrum of the analyzed signal.

## 3. Performed Experiments and Results

The experiments were carried out on the open-air, low-field (up to 0.2 T) MRI system E-scan Opera manufactured by the company Esaote S.p.A., Genoa, Italy [6]. This MRI system is located at the Institute of Measurement Science (IMS) in Bratislava, in the laboratory of the department of imaging methods. The piezoelectric vibration sensor was located in the scanning area of the MRI device; the microphone and the sound level meter were placed in its vicinity. The time course of the output voltage signals of the vibration sensor and the microphone was recorded, together with the measured noise SPL. The stored data were further processed to analyze the vibration and noise conditions by the energetic signal properties.

### 3.1. Automatic Measurement of Relative Sensitivity and Frequency Response of Vibration Sensors Suitable for Working in the Low Magnetic Field Environment

The preliminary measurements of the sensitivity and the frequency response of the vibration sensors were done using the vibration exciter ESE 201 on which the sensors were mounted. The Audio Precision System One (AP S1) with two programmable input and output channels was used as a signal generator for the exciter, as well as an input for simultaneous measurement of electrical signals from the sensors. The AP S1 output voltage for the exciter and the AP S1 input signal from the measured sensor were checked in parallel by the digital oscilloscope Rigol DS1102E. Three types of vibration sensors with the main element based on the piezoelectric principle were measured and tested:

- Cejpek SB-1 with a circular 27.5-mm brass disc designed primarily for pickup of a musical sound of a contrabass [15,16];
- RFT heart microphone device HM 692 with a 20-mm disc transducer comprising a piezoelectric element integrated in the aluminum metal cover with a 30-mm diameter; and
- SDT1-028K sensor (by Sensor Solutions—TE Connectivity) consisting of a rectangular piezo film element (with dimensions of 28.6 × 11.2 × 0.13 mm), together with a molded plastic housing, typically used in safety and industrial applications.

The vibration sensors were measured and compared by two types of parameters:

- Relative sensitivity at the reference frequency *f*_ref_ = 125 Hz; and
- Frequency response in the range of 20 Hz to 2 kHz at a chosen AP S1 output voltage for the vibration exciter (U_Ba0_ = 360 mV_ef_ = 1 V p-p).

**Figure 1:**
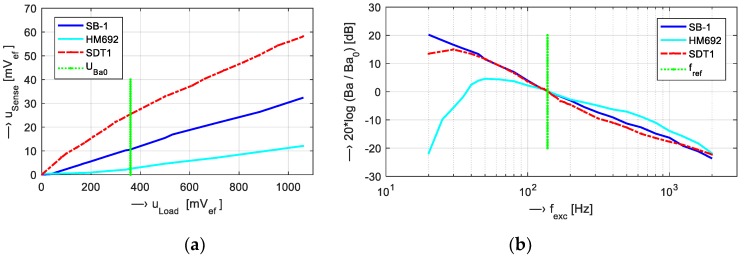
Graphs of (**a**) measured sensors’ sensitivities and (**b**) relative frequency responses [dB] in the range of 20 Hz to 2 kHz; *f*_ref_ =125 Hz; *Uexc_Ba0_* =360 mV; *B*_a0_ = {12.9 (SB-1), 2.45 (HM692), 26.0 (SDT1)} mV/m·s^−2^.

Dependence of all four sensors’ sensitivities on the excitation voltage can be seen in Figure 1a, and their frequency responses in [dB] in the range of 20 Hz to 2 kHz are depicted in Figure 1b.

### 3.2. Description of Main Measurement and Auxiliary Experiments

**Figure 2:**
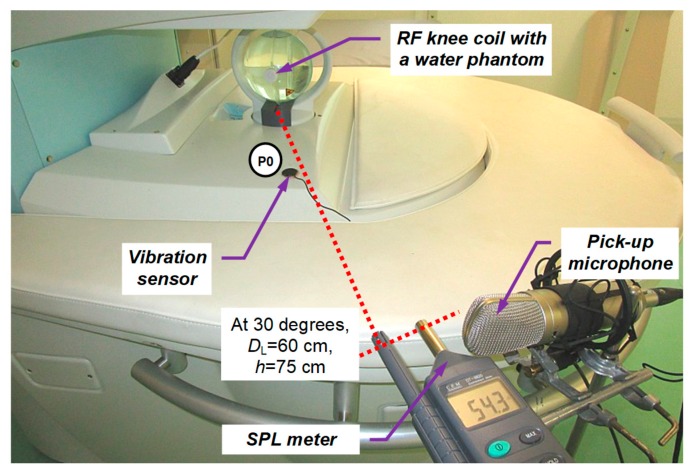
Arrangement photo of SPL noise measurement and parallel recording of noise and vibration signals of the open-air MRI device Opera using the testing phantom.

**Figure 3:**
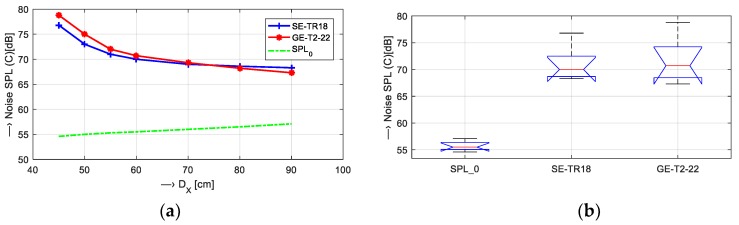
Mapping of the acoustic noise SPL at different distances D_X_ = {45, 50, 55, 60, 70, 80, and 90} cm from the middle of the scanning area of the MRI device for SE/GE sequences: (**a**) SPL values together with the background ones (SPL_0_), and (**b**) box-plot of their basic statistical parameters.

For all basic measurements, a plastic sphere-phantom of a 140-mm diameter filled with doped water [6] was placed inside the knee RF coil; see the arrangement photo in Figure 2. For acoustic noise SPL measurement in the MRI device vicinity, the multi-function environment meter Lafayette DT 8820 was used. Its distance from the center of the scanning area was 60 cm, its height from the floor was 75 cm (at the level of the bottom gradient coils), and it was oriented at 30 degrees from the left corner where the temperature stabilizer was placed. First, the noise SPL was mapped in the range of distances from 45 to 90 cm—the obtained values for Hi-Res SE and GE sequences are presented in Figure 3. As a result of the preliminary measurements, the SB-1 sensor was used to record the vibration signal from the solid surface of the plastic holder of the bottom gradient coils in the MRI scanning area—see position P0 in Figure 2. This figure also shows positions of the sound level meter (Lafayette DT 8820) and the dual diaphragm condenser microphone (Behringer B-2 PRO) on a stand with shock mounting for the noise SPL and signal pick up. The signals of the vibration and noise were routed through the Behringer Podcast Studio equipment by USB connection to PC. The signals with duration of about 15 s were sampled at 32 kHz and then processed in the sound editor program Sound Forge 9.0a.

The main experimental measurement was aimed at investigation of the impact of MRI scan parameters on the recorded vibration and noise. As presented in our earlier paper [17], only five types of scanning sequences are implemented in the investigated MRI device [6]:

1. SE sequences (11 sub-types);
2. GE sequences (9 sub-types);
3. Turbo (multi echo) sequences (4 sub-types);
4. 3D sequences (5 sub-types); and
5. Hi-Res sequences (8 sub-types).

In practice, two basic types of scan sequences are commonly used for non-invasive examination of human body parts by acquiring high-quality MR images in this type of MRI device:

- High-resolution SE/GE pulse sequences (Hi-Res); and
- 3D sequences to create 3D models of various biological objects.

**Figure 4:**
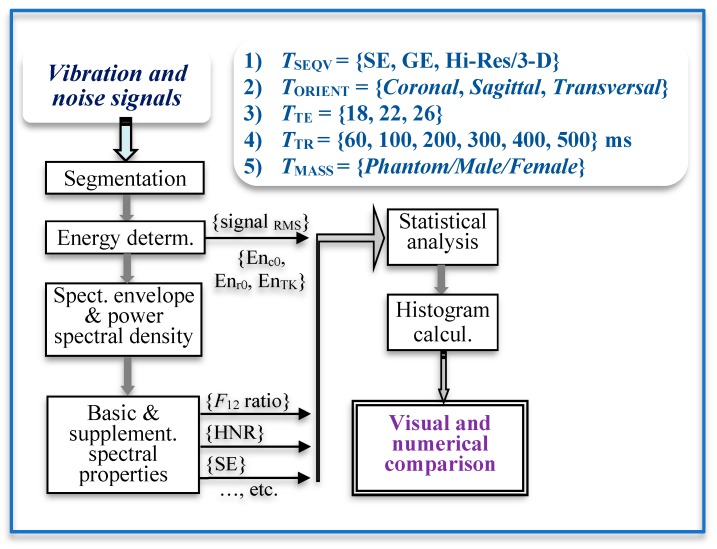
Block diagram of processing and comparison of vibration and noise signals.

The baseline measurement and recording of the vibration and noise signals were carried out during the execution of MR scan sequences typical for 3D imaging of the human vocal tract. Figure 4 shows how the energetic and spectral parameters of the picked-up vibration and noise signals are determined and processed.

**Figure 5:**
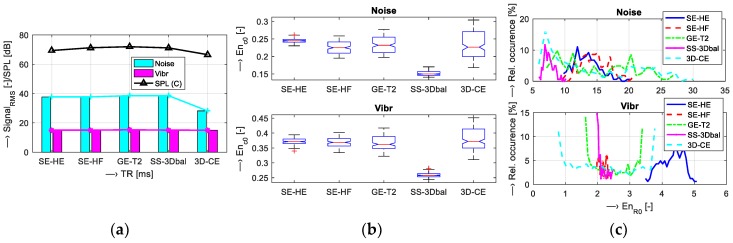
Comparison of energetic relations of vibration and noise signals for different sequence types—Hi-Res {SE-HE, SE-HF, GE-T2} and 3D {SS-3Dbal, 3D-CE}; (**a**) signal RMS together with SPL values; (**b**) bar-graphs of basic statistical parameters of *En*_c0_ values; (**c**) corresponding histograms for *En*_r0_ parameter. In all cases, a sagittal slice orientation was used.

**Figure 6:**
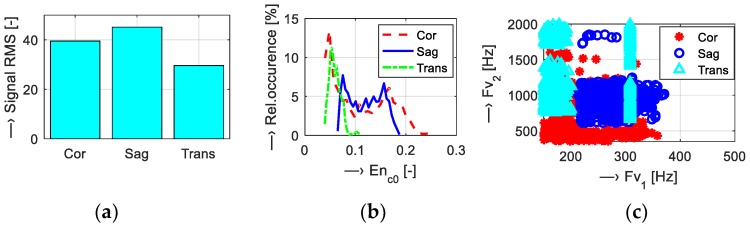
Visualization of vibration signal features for different slice orientations: {Coronal, sagittal, transversal}; (**a**) bar-graph of signal RMS values; (**b**) histograms of *En*_c0_; (**c**) mutual *F*_v1_/*F*_v2_ positions for Hi-Res SE scan sequences with TE = 18 ms and TR = 500 ms.

**Figure 7:**
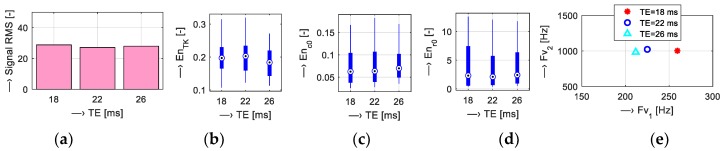
Visualization of vibration signal features for different TE times: {18, 22, 26} ms; (**a**) bar-graphs of signal RMS values and basic statistical parameters: (**b**) En_TK_; (**c**) En_c0_; (**d**) En_r0_; (**e**) mean mutual F_v1_/F_v2_ positions for Hi-Res SE-HF sequences (TR = 500 ms, sagittal orientation).

**Figure 8:**
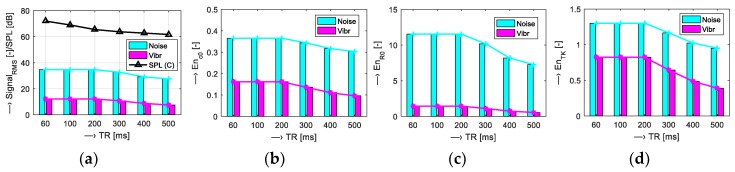
Visualization of energetic relations of vibration and noise signals for different TR times: {60, 100, 200, 300, 400, 500} ms; (**a**) signal RMS together with noise SPL values; (**b**) mean En_c0_; (**c**) mean En_r0_; (**d**) mean En_TK_; used Hi-Res GE-T2 sequences with TE = 22 ms, and sagittal orientation.

Scan parameters of five tested MR sequences (*T*_SEQV_ = {Hi-Res SE 18 HF, Hi-Res SE 26 HF, Hi-Res GE T2, SS-3Dbalanced, 3D-CE}) were set as shown in Table 1. Graphical comparison of energetic relations between the measured vibration and noise signals can be seen in Figure 5. Then, analysis of the influence of scan parameters on properties of the vibration and noise signals was executed for different parameters:

- Scan slice orientation *T*_ORIENT_ = {Coronal, Sagittal, Transversal}—results for energetic and basic spectral features are visualized in Figure 6;
- Echo time *T*_TE_ = {18, 22, 26} ms and repetition time *T*_TR_= {60, 100, 200, 300, 400, 500} ms—visualizations shown in Figure 7 and Figure 8; and
- Mass of the object/subject *T*_MASS_ = {Phantom/Male/Female} placed in the MRI scanning area (the testing phantom with the total weight of 0.75 kg or a head and a neck of a lying male/female person weighing approximately 80/55 kg was placed in the RF scan coil between the upper and lower gradient coils of the MRI device)—numerical comparison can be found in Table 2.

### 3.3. Parameters Determinig the Scan Sequence Duration and the MR Image Quality Factor

The final scanning time is practically defined by a chosen scan sequence and basic scan parameters (TR and TE). The default configuration [6] is often modified manually by changing these two parameters; however, other scan settings (number of slices, slice thickness, number of accumulations *N*_ACC_ of the free induction decay (FID) signal [13], etc.) also have influence on duration, as well as on the MR image quality. The main aim is to obtain final MR images with maximum quality factor (*Q*_F_) and minimum scan time duration (*T*_DUR_).

**Figure 9:**
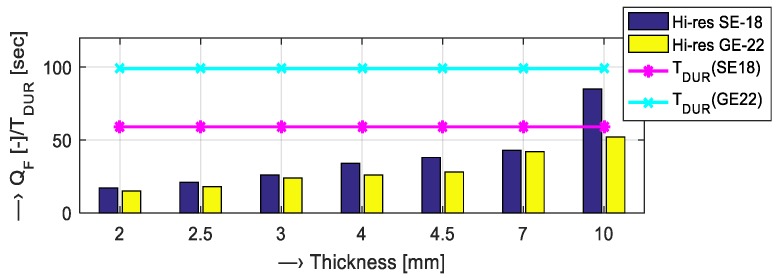
Influence of the thickness of the slice sample on the predicted quality factor and the time duration for the scan sequences Hi-Res SE18 HE and GE T2 22 (TR = 500 ms, *N*_ACC_ = 1).

**Figure 10:**
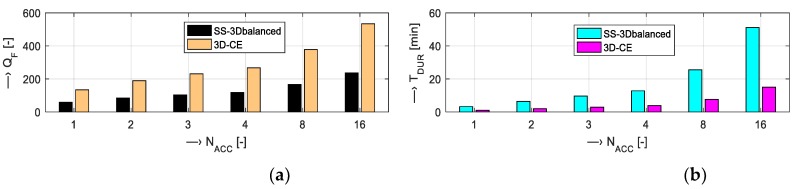
Influence of the number of FID signal accumulations on (**a**) the predicted image quality factor and (**b**) the time duration; analyzed scan sequences SS-3Dbalanced (TE = 10 ms, TR=20 ms, 3D phases = 24) and 3D-CE (TE = 30 ms, TR = 40 ms, 3D phases = 8).

Using the operating console of the Esaote Opera MRI device [6], we analyzed how the predicted MR image *Q*_F_ and the scan-sequence time duration are affected by the following scan parameters:

- Slice thickness {2, 2.5, 3, 4, 4.5, 7, 10} mm—predicted *Q*_F_ and *T*_DUR_ for the Hi-Res scan sequences SE18 HE and GE-T2 22 can be seen in a bar-graph in Figure 9;
- Repetition time *T*_TR_ = {60, 100, 200, 300, 400, 500} ms and the number of FID signal accumulations *N*_ACC_ = {1, 8, 16} for SE and GE types of Hi-Res sequences—numerical comparison of the obtained values is shown in Table 3 and Table 4; and
- Number of FID signal accumulations on the predicted *Q*_F_ and *T*_DUR_ for scanning sequences of 3D types—see the graphical comparison in Figure 10.

### 3.4. Subjective Evaluation by the Listening Test Method

Various emotions can be induced in humans by various auditory and/or visual stimuli. A set of six basic emotions is conventionally used by psychologists, although these discrete emotions can be projected into a two-dimensional plane with continuous values of pleasure and arousal axes [18]. The pleasure can change from negative (anger, sadness, fear, disgust) through neutral to positive (surprise, joy). The arousal can acquire values from low (passive emotions like apathetic sleepiness or boredom) to high (active emotion like frantic excitement) [19].

**Figure 11:**
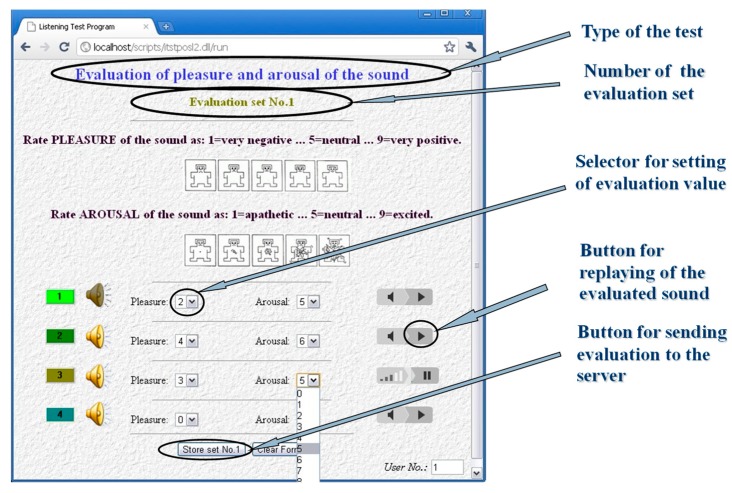
Screen shot of the user communication HTML page used to perform the listening test evaluation.

**Figure 12:**
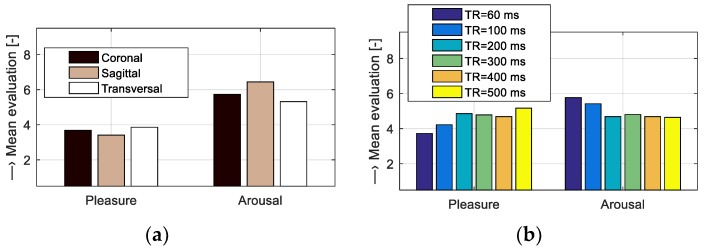
Bar-graph comparisons of evaluated pleasure and arousal parameters for MRI noises of (**a**) the Hi-Res SE sequence with different slice orientations, and (**b**) the Hi-Res GE sequence with different TR times.

**Figure 13:**
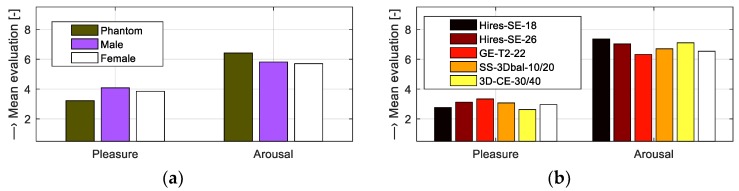
Bar-graph comparisons of evaluated pleasure and arousal parameters for MRI noises of the Hi-Res SE sequence for (**a**) different objects placed in the MRI scanning area and (**b**) different sequence types {Hi-Res SE-HE, Hi-Res SE-HF, Hi-Res GE-T2, SS-3Dbal, 3D-CE} using a water phantom; sagittal slice orientation in all cases.

Our subjective method of evaluation based on the listening test was focused on analysis of influence of vibration and noise generated by the gradient system of the MRI device on the human psyche. The aim of the test was to assess how listeners perceive different noises from emotional point of view. The server realization of the listening test “Evaluation of pleasure and arousal of sound” was operated at the internet site http://www.lef.um.savba.sk/Scripts/itstposl2.dll. This automatic application runs on the server PC as an MS ISAPI/NSAPI DLL script and communicates with the user within the framework of the HTTP protocol by means of the HTML pages—see an example of a screenshot in Figure 11. This system, designed originally for assessment of speech signal quality [20], serves at present the purpose of the MRI noise and vibration evaluation. Twenty-seven evaluators (8 women and 19 men) within the age range of 20 to 57 years participated in the listening test experiment open from 26 February to 20 March 2019. For compatibility with other emotional databases, like the International Affective Digitized Sounds [21], the listeners were instructed to use two parameters: Pleasantness (pleasure) and intensity (arousal), within the evaluation range of 1 to 9. The entire test consisted of 10 sets with 4 sounds each, so 40 different noises were evaluated. The listeners were allowed to play the audio stimuli as many times as they wished; low acoustic noise conditions and headphones were advised. The obtained evaluation results of the recorded MRI noises were presented by bar-graph comparisons. Figure 12 shows the emotional influence of the SE sequence with different slice orientations and the GE sequence with different TR times. Figure 13 gives the impact of the SE sequence for different masses in the scanning area, and finally, the effect of different scan sequences.

## 4. Discussion of Obtained Results

For 3D MR scanning of the vocal tract [1,8] during phonation, the frequency range of 25 Hz to 3.5 kHz covering pitch and formant frequencies must be included in the recordings. A preliminary analysis of the properties of the vibration sensors suitable for measurement in the low magnetic field environment confirmed a general presumption of inverse relationship between the diameter of the used sensor and the minimum frequency of vibration picked up from the measured surface. At the same time, the maximum frequency, as well as the sensitivity, were decreased in the massive aluminum microphone capsule of the phonocardiographic sensor HM692—its frequency response in Figure 1b shows a local maximum at about 50 Hz, which makes it useless for our purpose. The piezo film vibration sensor SDT1 has the best sensitivity but its frequency response shows higher non-linearity in comparison with the sensor SB-1. In addition, the vibration sensor SB-1 has the highest low frequency sensitivity, so it was finally chosen for all next recording and measurement experiments.

The acoustic noise intensity was measured at several distances from the center of the scanning area, beginning with 45 cm because, for shorter distances, the magnetic field inhomogeneity due to interaction with metal parts of the sound level meter causes the display of a warning message on the MRI control console and interruption of further scanning to prevent failure of the MRI device [6]. At distances longer than 90 cm, the measured sound level approached the background noise level of the temperature stabilizer. The results of these measurements for high-resolution SE and GE pulse sequences can be seen in Figure 3. All the following measurements were carried out at the distance of 60 cm.

Results of the first comparison of energetic relations of vibration and noise signals are documented in Figure 5. Small differences were found for all sequence types, but the 3D-CE sequence produced the noise with minimal intensity expressed by the signal RMS parameter and SS-3Dbal calculated using the *En*_c0_ parameter. Further investigation was aimed at the influence of the choice of slice orientation on the energy of the produced vibration and noise signals. The graphs in Figure 6 show maximum energy in the sagittal plane and minimum energy in the transversal plane. Sagittal orientation was used in the remaining experiments to explore the worst case. In accordance with our previous research [15,16,17], the current experiments confirm the influence of TR and TE times on the vibration and acoustic noise properties. Prolongation of the TE time was accompanied by a slight lowering of the final signal energy with decreased first dominant frequency *F*_V1_, as documented in Figure 7. Higher TR parameter determining the fundamental frequency *F*_V0_ had greater influence on lowering the signal energy, especially for TR = 400 and 500 ms, as can be seen in Figure 8. The obtained vibration signal energy was higher for the water phantom than for the lying person (see Table 2), due to partial attenuation of vibration pulses by higher effective weight pressing on the bottom plastic holder of the gradient coils. At the same time, the noise signal energy had its maximum for the lying male person because of higher noise induced by vibration of the upper gradient coils that are not loaded by the tested object. From a physical point of view, larger volume of a person in the MRI scanning area needs higher electrical currents in the gradient coils, causing higher Lorentz forces and greater vibration of upper gradient coils for the examined human body in comparison with the 140-mm diameter spherical testing phantom.

Preliminary analysis shows positive influence of increased slice thickness on the predicted MR image *Q*_F_ for both SE and GE Hi-Res scanning sequences—compare Figure 9. The effect of TR and *N*_ACC_ on *T*_DUR_ and predicted *Q*_F_ of the executed scanning sequence is shown in Table 3 and Table 4. While the increased repetition time caused only slightly greater overall time duration, the number of accumulations affected the final time duration very much. This applies for both types of Hi-Res sequences: Increasing *N*_ACC_ from 1 to 16 resulted in about 4 times greater *Q*_F_ and about 15 times greater *T*_DUR_. The same trend was observed for increased TR, its change from 60 to 500 ms caused about 7 (1.9) times greater *Q*_F_ and about 7 (8) times greater *T*_DUR_ for SE (GE). This course is valid also for two 3D scanning sequences, as seen in Figure 10. Here, the increased time duration was influenced also by the number of 3D phases being an equivalent to the number of slices (selected by the slice thickness) in Hi-Res sequences.

## 5. Conclusions

The results of the performed measurements help in precise description of the process of mechanical vibration excitation and the acoustic noise radiation in the scanning area and vicinity of the MRI device [22,23]. Comparison with a similar low-field MRI tomograph can be used for optimization of acoustic noise suppression in parallel with speech recording applied in 3D modeling of the human vocal tract [8]. The main usage of the obtained results is expected in experimental practice, when the used scan sequence and its parameters must often be changed depending on the person being tested. So far, our MRI experiments with vocal tract scanning were realized only on healthy people—typically, the trained MRI operators from the IMS and the authors of this paper themselves. In future, we plan cooperation with a medical center certified for work with patients. Then, our MRI equipment can be used for monitoring progress in treatment of diseases of the vocal cords and the vocal tract. For other users of this type of an open-air MRI device (in our country or in neighboring countries), we can only recommend choice of proper MR sequences, as well as modification of scan parameters as a compromise between noise and vibration exposition of an examined person and obtaining high-quality MR images.

We are aware of the limits of this study—at present, there exist a lot of modern MRI devices enabling parallel processing of MR images, thus rapidly shortening the necessary scanning time duration and subsequently minimizing the negative noise and vibration effect on patients. As the gradient coils are the integral part of the whole MRI equipment [6], we as users cannot make any changes to them; only external RF coils can be developed and connected to the system [24]. For the same reason, any system interference (e.g., adding new materials for damping of mechanical vibrations) cannot be recommended or advised as the output of this study.

Combination of the vibration and acoustic analysis can also be used in fault diagnosis of vibrating mechanical systems [25]. In our case, abnormal vibration and acoustic noise could reflect some fault in the MRI sequence generation. The performed measurement can also be reproducible on other types of low-field MRI devices; however, in the case of whole-body tomographs, picking up the vibration, as well as recording and measurement of the acoustic noise, are more difficult. In this case, the results obtained with an open-air device can be applied. A comparison study, including a discussion to this problem, has already been published in our last journal article [26].

The maximum noise SPL of about 78 dB (C) was measured at the closest distance from the central point of the MRI scanning area (45 cm), while the GE scan sequence with short TE and TR was running and the sagittal orientation was set. Although special hearing protection aids are not unconditionally necessary, ear plugs or ear muffs can contribute to the comfort of the examined person. If the scanned part of the human body inserted between the upper and the lower gradient coils is farther from the head, the ears are exposed by much lower noise. However, the absolute values of the vibration and noise signal RMS depend on signal amplification by the recording device, microphone directional pattern, and vibration sensor placement. The measurement of the noise itself is affected by a chosen measuring range (Lo/Hi) and a weighting filter (A/C) of the SPL meter. Therefore, direct comparison with the three relative energetic parameters calculated by Equations (3) and (4) is not possible. The scanning time duration depends on the chosen number of slices and their thickness. For 3D and Hi-Res sequences, it is usually lower than 15 minutes (typically about 3 to 5 minutes), so the vibration and noise exposition of the examined person does not mean a threat to his/her health. If more detailed MR images with higher quality factor *Q*_F_ must be done (e.g., scans of particular parts of the human brain, the eye, the middle and inner ear, etc.), the patient will be exposed to the vibration and acoustic noise for a longer time *T*_DUR_ (exceeding half an hour), which might be followed by a rather great physiological and psychological stress. Only urgent cases justify using these scan parameters in medical practice. 

Our next research will be focused on detailed investigation of the negative influence of the generated acoustic noise on the physiological and psychological state of the examined person lying in the scanning area of the low-field MRI device. This negative influence on a human body can be monitored by measuring the blood pressure (BP) and heart rate (HR), as the stress is manifested by changes in the bloodstream. These changes can also be successfully determined directly from MR images of different parts of the human body—typically from the veins in the handbreadth area [27]. In addition, we plan parallel measurement of BP and HR parameters of the currently scanned person during execution of the whole MR scan sequence. For this purpose, we will apply photo-plethysmography (PPG) using optical sensors for non-invasive retrieval of vital information about the cardiovascular system from the skin surface [28,29]. Variations in the photo-detector signal are related to changes in the blood volume inside the tissue. Signal filtering and further processing will be necessary to obtain a clean PPG waveform, which can then be used to derive the instantaneous heart rate. This optical-based approach is fully in compliance with the requirements for sensors working in the magnetic field environment where RF and electromagnetic disturbances are present.

## Figures and Tables

**Table 1:** Basic scan parameters of used MR sequences.

Type	Name of Sequence	TE (ms)	TR (ms)	FOV	Matrix Size
Hi-Res	SE 18 HF	18	500	250 × 250 × 200	256 × 256
Hi-Res	SE 26 HF	26	500	250 × 250 × 200	256 × 256
Hi-Res	GE T2	22	60	250 × 250 × 200	256 × 256
3D	SS 3D balanced	5	10	200 × 200 × 192	200 × 200
3D	3D-CE	30	40	150 × 150 × 192	192 × 192

**Table 2:** Comparison of mean energy values of vibration and noise signals for different objects placed in the scanning area of the MRI device.

Subject Type ^1^	Vibrations (SB-1)				Noise (B2-Pro)			
	RMS	*En*_TK_	*En*_c0_	*En*_r0_	RMS	*En*_TK_	*En*_c0_	*En*_r0_
Water phantom	34.6	4.69	0.0380	24.0	20.1	4.05	0.0255	8.5
Female	28.7	4.93	0.0402	16.6	23.2	4.19	0.0286	10.6
Male	26.8	4.96	0.0404	14.4	25.5	4.51	0.0328	15.9

^1^ Used Hi-Res SE-HF scan sequences with TE = 18 ms, TR = 400 ms, and sagittal orientation.

**Table 3:** Dependence of the MR image quality factor *Q*_F_ and the scanning time duration *T*_DUR_ [min:sec] on TR and *N*_ACC_ parameters; used Hi-Res SE26 HF sequence, slice thickness = 4.5 mm.

*N*_ACC_ [–]	Parameters	TR [ms]					
		60	100	200	300	400	500
**1**	*Q*_F_ [–]	6	12	23	32	38	42
	TDUR [min:sec]	0:09	0:14	0:25	0:36	0:48	0:59
**8**	*Q*_F_ [–]	16	33	66	90	107	500
	TDUR [min:sec]	0:57	1:33	3:04	4:34	6:04	7:35
**16**	*Q*_F_ [–]	23	47	93	127	151	168
	TDUR [min:sec]	1:51	3:03	6:04	9:05	12:06	15:07

**Table 4:** Dependence of the MR image quality factor *Q*_F_ and the scanning time duration *T*_DUR_ [min:sec] on TR and *N*_ACC_ parameters; used Hi-Res GE-T2 22 sequence, slice thickness = 4.5 mm.

*N*_ACC_ [–]	Parameters	TR [ms]					
		60	100	200	300	400	500
**1**	*Q*_F_ [–]	15	18	24	26	27	28
	T_DUR_ [min:sec]	0:14	0:24	0:42	1:00	1:20	1:39
**8**	*Q*_F_ [–]	42	52	67	74	77	79
	T_DUR_ [min:sec]	1:35	2:37	5:12	7:46	10:20	12:55
**16**	*Q*_F_ [–]	59	74	95	104	109	112
	T_DUR_ [min:sec]	3:08	5:11	10:20	15:29	20:38	25:47
